# Bridging the gap between basic research and commercial application at scale for in vitro bovine embryo production

**DOI:** 10.1590/1984-3143-AR2026-0080

**Published:** 2026-09-18

**Authors:** Rebecca L. Krisher

**Affiliations:** 1 Department of Animal Science, The Pennsylvania State University, University Park, PA, USA

**Keywords:** in vitro fertilization, embryo transfer, translational pipeline

## Abstract

The rapid expansion of in vitro embryo production (IVP) in cattle has established this technology as a major driver of genetic progress and production efficiency. Despite widespread adoption, IVP systems remain inherently inefficient, with substantial losses occurring from oocyte to blastocyst and variable outcomes following transfer. At the same time, significant advances in the understanding of oocyte competence and embryo biology have occurred. However, this knowledge has not consistently translated into improvements in commercial embryo production. The purpose of this review is to examine the gap between basic discovery and field application in bovine IVP and to define the biological and operational factors that limit successful translation at scale. A central constraint is the fundamental difference between systems optimized under controlled laboratory conditions and those required to perform consistently across diverse donor populations, laboratory environments, and high-throughput workflows. Biological variability, metabolic regulation, and the complexity of embryo physiology intersect with operational feasibility, including scalability, cost, and workforce constraints, to limit adoption of new approaches. A structured translational pipeline, from discovery through proof of concept, development, and implementation, is required to align mechanistic insight with commercial realities. Successful translation depends on integrating biological understanding with system robustness, evaluating outcomes beyond early developmental endpoints, and aligning research and industry priorities throughout the development process. Strengthening the collaboration between research and industry is essential for continued innovation and growth of bovine IVP systems.

## Introduction: The rise of large-scale in vitro embryo production in bovine

The rapid expansion of in vitro embryo production (IVP) in the bovine industry underscores its value as a driver of genetic progress. At the same time, increasing global demand for animal protein, coupled with constraints related to sustainability and resource use, places pressure on production systems to deliver greater efficiency. Despite widespread adoption, IVP remains inherently inefficient. A substantial portion of this inefficiency reflects both the low conversion of oocytes to high-quality blastocysts and the variable developmental competence of those embryos (Demetrio et al., 2020). Addressing these limitations will require improvements in culture systems that support embryo physiology more effectively, resulting in embryos with enhanced viability, cryotolerance, and capacity to establish and maintain pregnancy, while ultimately producing healthy offspring (Ferre et al., 2020; Hansen, 2024; Crowe et al., 2025).

In the last twenty years, basic researchers have discovered a great deal about the molecular mechanisms regarding control of oocyte competence and embryo viability in cattle (Fair and Lonergan, 2023). During this same period, commercial transfer of in vitro derived (IVP) bovine embryos has rapidly increased across the globe (Anderson, 2023; Lonergan, 2024; Viana 2024). More than two million IVP cattle embryos were produced in 2024, a number seven times that of in vivo derived (IVD) embryos (Viana, 2025). This number represents a staggering adoption of reproductive technology at scale by the cattle industry. Embryo technologies allow rapid genetic improvement that has transformed animal efficiency. However, in vitro culture conditions can have detrimental effects on embryo, fetal, and neonatal viability (Lonergan, 2024; Lonergan and Fair, 2014). The urgent demand for high quality, consistent embryo production has highlighted the inherent variability in blastocyst development, establishment of pregnancy, and pregnancy outcomes of commercial IVP systems. Thus, while basic research has advanced our understanding of oocyte and embryo development, translating this knowledge into robust standard operating protocols (SOP) at scale remains a challenge (Hansen, 2023). Little attention has been given to the translational process from basic and even applied laboratory research to functional large scale commercial IVP systems producing thousands of embryos each week for transfer. Bridging this gap between discovery and application is central to continued success in commercial bovine embryo transfer (ET) businesses.

Embryo transfer is widely accepted as an important tool for genetic improvement (Hansen, 2023). Oocytes can be collected from pre-pubertal and pregnant females of high genetic merit and fertilized with sex sorted semen from sires of high merit. Embryo transfer, by increasing the efficiency and speed of genetic gain, has a significant impact on the sustainability of animal protein production. Life cycle assessments, the standard method to evaluate environmental impact, have demonstrated that genetic improvement reduces environmental impact via reduced emissions intensity in beef and swine production (Putman et al., 2023; Thoma et al., 2024). In vitro fertilization and embryo transfer (IVF-ET, or IVP) can also improve fertility during heat stress, facilitate the production of high value beef animals from dairy cows, and is a critical component in technologies such as gene editing, nuclear transfer, and gamete generation from stem cells (in vitro breeding) (Hansen, 2023). Finally, embryo transfer is recognized as the most biosecure way to move genetics across the globe (Funnell et al., 2024). Compelling reasons such as these help explain the rapid growth in cattle IVP globally; the number of commercial cattle embryos recorded by the International Embryo Technology Society has increased more than ten times since 1990. More than 25 million embryos were transferred during this period. In 2017, more IVP than IVD embryos were recorded for the first time. Since then, the rapid increase in IVP embryos specifically has driven industry numbers. Brazil was largely responsible for early IVP adoption, producing 75% of the world’s IVP embryos in 2012. Currently, more than 97% of Brazil’s embryos are IVP, although that country’s embryo numbers are considered to be vastly underreported. Today, the US produces over 50% of the world’s IVP embryos, equating to more than 1 million IVP embryos annually (Viana, 2025). The number of IVP embryos produced has grown by over 10% annually for 3 of the last 4 years.

To meet the large, and growing, demand for IVP embryos (Viana, 2025), embryo production laboratories have been created to work with huge numbers of oocytes and embryos every single day including weekends and most holidays. This is a vastly different scenario than a research laboratory that may work with 300-400 oocytes a day for about two days a week. The protocols and processes in a commercial lab at this scale must be simple, streamlined, and translatable to a workforce with variable skill levels at entry. A commercial lab must produce embryos of consistent quality that will reliably produce a high pregnancy rate with few fetal or neonatal complications. A research lab rarely studies any effect after blastocyst development. The vast differences in these two bovine IVP systems contribute to the limited implementation of research findings to commercial embryo production.

## The translational pipeline

Addressing this gap requires a structured process to move discoveries from controlled settings into commercial systems. In order to translate basic discovery into real world products or processes, there must be an established pipeline that adapts and validates new knowledge for commercial applications, similar to drug discovery (Hughes et al., 2011). In bovine IVP, successful translation requires integrating mechanistic biological insight with the operational constraints of commercial embryo production. A robust process is essential for technological advancements to occur. This process is often referred to as the product development lifecycle, and includes the following phases: discovery, proof of concept (POC), early and late development, and finally launch. This process allows mechanistic ideas to be transformed into commercial solutions with structured risk management and clear go/no-go milestones. The commercial stakeholders have ever greater input and ownership as a product moves through this pipeline. Many academic labs operate solely in the discovery phase and are unaware of this larger process. To be successful, collaboration between basic research labs and commercial companies is critical to ensure that end user needs are aligned with experimental plans from the beginning.

Discovery is the initial phase that is focused on defining the problem, understanding mechanisms, conducting exploratory research, and confirming biological relevance under controlled laboratory conditions. This phase also ensures that all stakeholders are aligned on potential outcomes and business impact. Most ideas do not progress out of discovery to POC. When and if initial feasibility is demonstrated in discovery phase, then the product or process moves to POC where additional technical feasibility and viability testing, validation, and optimization occurs. To exit POC, statistical evidence must suggest that the product is reasonably likely to succeed and at a reasonable cost. In early development, the new product or process is adapted to the commercial production environment, and preliminary field tests occur to investigate safety and efficacy. In late development the product is refined and scaled up, and any necessary regulatory approval is obtained. Any bottlenecks in supply chain, process, or scalability are determined in these two phases. Finally, launch is reached and the product is deployed. Understanding how basic discoveries move through the product development lifecycle to reach eventual adoption in the embryo transfer industry is essential for both basic researchers and commercial businesses to avoid costly mistakes and frustrations and maintain trust. Without a structed process, valuable discoveries may languish in journal articles, money may be wasted pursuing solutions to problems the industry does not find important, or the solutions found may be too costly or impractical to be implemented.

## Translating oocyte and embryo metabolism into in vitro culture systems

These sources of variability place additional demands on culture systems, particularly in how embryo metabolism is supported in vitro. A central limitation that remains unresolved is that in vitro conditions support blastocyst development, but do not reliably support in vivo-like physiology or downstream viability (Driver et al., 2012, Ming et al., 2024). The historical progression from undefined co-culture systems to chemically defined media (e.g., SOF, CR1AA, KSOM) reflects two dominant design philosophies—recapitulating the reproductive tract environment versus providing a permissive nutrient landscape from which the embryo selects (Zander-Fox et al., 2023; Krisher and Herrick, 2024). Despite these advances, efficiency gains have been modest, with substantial attrition from oocyte to transferable embryo, from transfer to pregnancy, and from initial pregnancy diagnosis to healthy calf. This would suggest that current systems fail to fully support the biological requirements of the embryo and highlights that developmental competence is tightly linked to metabolic regulation rather than simply blastocyst production (Leese et al., 2008). Embryos exhibit considerable metabolic plasticity, but this flexibility often reflects adaptation to suboptimal conditions rather than optimal function (Lonergan and Fair, 2014). Concepts such as the “quiet embryo” hypothesis and alternative metabolic models emphasize that viability is associated with appropriate pathway utilization and maintenance of redox balance, not simply reduced or increased metabolic activity (Leese et al., 2008; Leese et al., 2022; Krisher and Prather, 2012). In vitro culture conditions, then, must translate fundamental knowledge about oocyte and embryo metabolism into culture systems that support successful IVP.

Although that task seems straightforward, it is in fact inherently complex. Multiple metabolic pathways, including glucose, amino acids, fatty acids, mitochondrial activity, and redox potential require delicate orchestration. To complicate matters, many of these metabolic pathways are intricately linked (Sutton-McDowall et al., 2012; Paczkowski et al., 2014; Herrick et al., 2020). Stress must also be minimized while working within a system that to date is entirely unlike the physical environment in vivo (Gebremedhn et al., 2026). An evaluation of overall metabolic activity rather than focus on a single substrate or pathway is more likely to result in successful translation of metabolism to culture medium.

Metabolomic analyses of nutrient composition of media following individual embryo culture suggest that embryos utilize only a fraction of available nutrients (Krisher et al., 2015; Herrick et al., 2016). Surprisingly, bovine embryo development was not affected when nutrient concentrations in culture media were reduced by as much as 75% (Herrick et al., 2020; Santos et al., 2021). Blastocyst development and quality could be significantly improved under these reduced nutrient conditions by promoting lipid metabolism (Pasquariello et al., 2023). The transcriptome of embryos grown in reduced nutrient conditions suggests these conditions support metabolic activity and developmental potential that is more like in vivo embryos (Ming et al., 2024). Collectively, these findings underscore an essential difference between the ability to produce blastocysts in vitro and the ability to produce embryos in vitro that are physiologically normal, reinforcing the need to reframe culture system design around fundamental embryo biology as a prerequisite for scalable, high-quality embryo production.

Our reduced nutrient work is a good example of how difficult it is to translate exciting basic discoveries into commercially viable advances. Although initial feasibility was demonstrated in these experiments—discovery phase—we were unable to carry out the needed post transfer viability and calf health studies required to move forward. Later, commercial business needs did not align with further exploration of this work, although anecdotally we spoke to commercial users of reduced nutrient media that report no negative or positive impact on pregnancy rate but instead use the system to reduce reagent costs. It is possible that further intentional development of the reduced nutrient system in carefully designed POC and early development phases might further develop this media to improve outcomes. Ultimately, stakeholder priorities must align with interesting basic discoveries to support progression through the product development lifecycle. A more successful example of such alignment is maturation medium that did not require pre-equilibration or gassing with CO_2_ after oocytes were added to maintain pH in the field. We had developed and tested CO_2_ free maturation medium in the research lab, but it was only when it became an urgent priority for the business that alignment was obtained, at which point it quickly moved through POC, early and late development, and we were able to scale production successfully to launch the product.

Ultimately, business interest in significantly improving existing technology or filling a demonstrated need must support further testing, and businesses must bring knowledge of implementation roadblocks and consumer costs before development can move forward. Thus, collaboration between basic scientists and industry is essential for successful translation to occur. Even when all these things align, successful implementation often hinges upon proper education, training, and gaining trust of the end users. This is understandably true for embryologists as well, because they are highly skilled and experienced in embryo production systems, and any change in the system comes with risk.

## Scaling IVP: From the research lab to industry production

While many experimental approaches improve embryo development under controlled laboratory conditions, their translation to commercial IVP systems is not straightforward.

Large-scale production environments routinely process hundreds of oocytes daily derived from donors with substantial variability in age, physiology, genetics, and reproductive history. Compounding this biological variation are operational factors, including technician-dependent workflows, laboratory environment, and logistical constraints that influence culture conditions and embryo handling. As a result, interventions that demonstrate efficacy in tightly controlled settings often do not deliver consistent outcomes at scale. A central challenge, therefore, is not simply optimizing embryo development under ideal conditions, but developing systems that are robust enough to be beneficial under both biological and operational variability, which remains a defining objective of translational embryology.

Differences in oocyte source and handling represent the first point where research and commercial IVF laboratories typically diverge (Hansen et al., 2010; Merton et al., 2013). In a research laboratory, oocytes are often obtained from a single abattoir that focuses on a specific type of animal, cull dairy cows or feedlot beef heifers, for example. Biological variation certainly exists in this system, but experimental designs are often in place to reduce variation for robust statistical analyses. Oocytes may be obtained from follicles of predetermined sizes, and only the best quality oocytes selected for maturation. Oocyte recovery and selection take place in a carefully controlled environment, and oocytes are placed immediately into maturation medium in dishes within a calibrated CO_2_ incubator that maintains pH in a tight range. Embryologists are typically graduate students that are working on individual experiments with small numbers of oocytes and treatments.

In contrast, commercial donors are selected for oocyte recovery typically based on genetic merit, regardless of previous IVF performance, and the lab must work with whatever oocytes are collected. The greater variability of animals in a production setting due to housing, nutrition, genetics, disease, or other factors may overwhelm the positive effect seen in discovery. Commercial donors may be prepubertal heifers or even calves (Currin et al., 2017; Kulus et al., 2026; Moura et al., 2025). Most commercial labs have some selection criteria to avoid placing dead or dying oocytes into maturation (Demetrio et al., 2022), although this varies across commercial labs and is typically less stringent than the criteria used for research. Oocytes are typically collected on-farm and must be physically transported to the central IVF lab, which necessitates maturation in tubes that are typically gassed with CO_2_ to maintain pH prior to transport. This often leads to varying levels of pH in the maturation tubes. In commercial laboratories, because of the different geographical location of farm and IVF lab, there is often a position called a ‘searcher’ whose job it is to collect, select, and processes oocytes from many donors on the farm before sending them to the lab. Searchers can be highly experienced, although this position is sometimes used as a training ground for IVF lab positions.

After maturation, the differences continue in the choice of semen for fertilization (Sosa and Ortega, 2025). A research laboratory typically will test several collections of conventional frozen semen to find a bull that supports good embryo development. A research lab may spend the time to characterize the best sperm and heparin concentration of that ejaculate to further optimize results. Fertilization usually takes place in drops, with an equal number of oocytes and sperm per drop. There are typically a small number of dishes to be prepared or manipulated on any given day. Commercially, the client chooses the semen, both conventional and sexed semen are used, and there is no opportunity for testing or optimization. The embryologist may have some information about semen performance in IVF, or none. Clients may ask to have a donor’s eggs fertilized by the same bull or split between bulls. Each donor must be carefully tracked throughout the process, and eggs from different donors must be maintained separately, leading to large numbers of dishes. In addition, fertilization drops contain variable numbers of oocytes from each donor.

After fertilization, presumptive zygotes are placed into culture drops for development to blastocyst. Culture media and SOPs may differ substantially, ranging from uninterrupted culture to systems requiring media replacement or embryo movement, approaches that are feasible in research settings but difficult to implement at commercial scale. Both preparing the number of dishes required, as well as physically moving the embryos, is too time-consuming and labor intensive for large scale commercial labs. Research laboratories typically culture embryos to day 7 or 8 before performing a wide array of analyses depending on the aims of the experiment. Commercially, embryologists may need to evaluate embryos on D5 to gauge recipient needs for ET on day 7. To accommodate working recipients on the farm, embryos may be transferred early on D7, while those that develop later might be cryopreserved, separating good quality blastocysts into two populations.

The scale of commercial IVP introduces additional logistical constraints. High volumes require continuous daily processing across maturation, fertilization, culture, cryopreservation, and ET, often with overlapping workflows. Efficiency is critical. As a result, timing is less precise than in research settings, with batching of fertilization and culture steps and broader time windows. These constraints place additional demands on system robustness and consistency. Supplements that improve embryo development in the research laboratory may not be stable or may require handling that is not practical in a commercial laboratory. Recognizing and addressing these multiple sources of biological and operational heterogeneity are essential for translating laboratory discoveries into protocols that perform reliably in large-scale embryo production systems.

There are some similarities between research and commercial systems. Both rely on the standardized IETS embryo grading system and operate with a transient workforce, making training and adherence to SOPs critical. Without proper training and a period of observation, protocols often change or get sloppy over time, resulting in different processes within a lab or between labs within a company, and lack of knowledge about current practices which can hamper troubleshooting efforts. Across both settings, embryologists are highly skilled and committed to producing high quality embryos, although the consequences of poor outcomes are substantially greater in commercial systems. Together, these differences illustrate that successful translation requires systems designed not only for biological performance, but for consistency under the biological variability and logistical constraints of scale in commercial IVP.

## Principles of translational embryology

Taken together, these observations define a set of principles that govern successful translation in bovine IVP. Progress in culture media improvement is most effective when media systems are grounded in a mechanistic understanding of oocyte and embryo biology, including metabolic requirements, mitochondrial function, redox balance, and interactions with surrounding somatic cells. Defining the biological basis of developmental competence enables a more robust and rational approach to culture system design across biological heterogeneity, one that supports normal embryo physiology to maximize both blastocyst yield and quality. In addition to biological variability, scale is also critical for translation. Many researchers do not fully appreciate that success depends as much on operational feasibility as biological performance. Media, culture systems, and handling procedures must be robust, cost effective, logistically feasible, and practical to implement under real world conditions. Approaches that are overly complex, costly, or sensitive to variation rarely achieve adoption, even when performance is strong in controlled settings. Finally, blastocyst development is a useful indicator, but it does not capture downstream outcomes. For translation to be successful, evaluation must extend beyond early endpoints to include cryotolerance, pregnancy success, and offspring health (Amaral et al., 2022; Gebremedhn et al., 2026; Zolini et al., 2019). Academic laboratories are well positioned to define underlying biological mechanisms and generate new concepts, while commercial IVP laboratories provide the scale, constraints, and validation required for implementation. Translation of discoveries and progress in bovine commercial IVP depend on close collaboration between the two. Together, these principles emphasize that effective translation in bovine IVP requires integration of mechanistic understanding of embryo biology with the practical constraints of large-scale production systems. Approaches that account for biological variability, operational feasibility, and long-term developmental outcomes, in which research and industry are aligned on objectives, are more likely to move successfully from discovery to field application (Figure 1).

**Figure 1:**
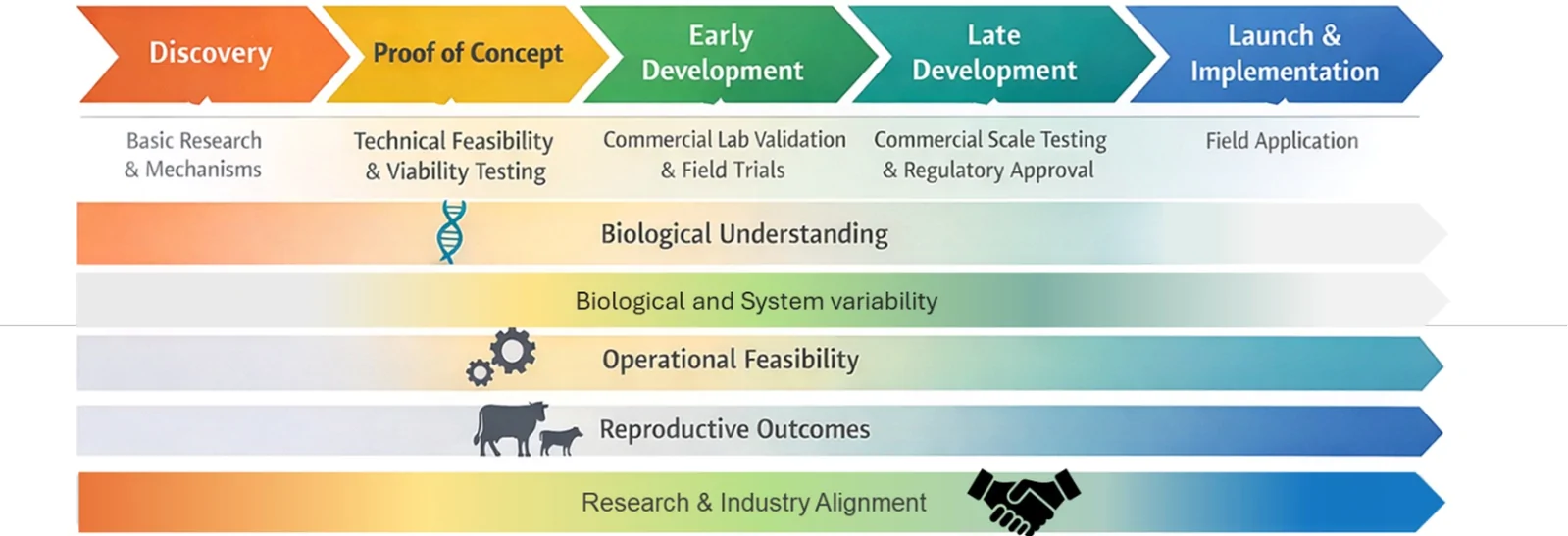
Principles of translational embryology in bovine in vitro embryo production.

Increased commercial adoption of bovine IVP arises through a translational pipeline that progresses from basic discovery science to field implementation in commercial embryo production systems. Successful movement across this pipeline is supported by several guiding principles: mechanistic understanding of oocyte and embryo biology, recognition of biological heterogeneity among donor animals, development of robust and scalable protocols, evaluation based on long-term developmental outcomes, and collaboration between research laboratories and industry partners. Integrating these principles appropriately within the translational pipeline increases the likelihood that discoveries in reproductive biology will translate into technologies that improve embryo production efficiency at commercial scale. 

## Future directions

Emerging technologies will play an important role in advancing bovine IVP and narrowing the gap between discovery and application. These include cutting edge technologies such as single embryo metabolomics, non-invasive embryo genotyping and quality assessment, artificial intelligence to design culture medium based on metabolomics and predict embryo viability, automated IVF, and precision IVF protocols for specific breeds or donor categories. While these technologies would meaningfully advance in vitro embryo production, they are likely to remain financially out of reach for most bovine commercial breeding and embryo companies well into the future. However, human IVF is driving many of these advances and there will be opportunities to integrate the knowledge generated into bovine IVP over time. Integration of these emerging technologies with mechanistic biology generated by research laboratories will enable new ways to improve and scale bovine IVP, allowing the industry to continue to grow.

## Conclusions

Continued progress in bovine IVP will depend on more effectively linking research discoveries with the realities of large-scale embryo production. While substantial advances have been made in understanding oocyte and embryo biology, these insights have not consistently translated into improvements in efficiency, consistency, or downstream outcomes at the commercial level. A central constraint remains the gap between systems optimized under controlled laboratory conditions and those required to perform reliably across diverse donor populations, laboratory environments, and production scales. Bridging this gap will require intentional integration of mechanistic biology with operational feasibility, as well as alignment between academic and commercial priorities throughout a well-defined development process from discovery to launch. Approaches that are grounded in biology, robust to variation, and evaluated based on meaningful production outcomes are most likely to drive sustained improvement. Strengthening the connection between discovery and application is essential for continued innovation, broader adoption, and long-term growth of bovine IVP.

## Data Availability

No research data was used.
